# Life Insurance Associations

**Published:** 1880-01

**Authors:** 


### LIFE INSURANCE ASSOCIATIONS.

---

In contrasting a few items of information derived from an experience of several years as medical examiner and member of some of the older life insurance companies, with what has been learned of the operations of two or three of the more recent purely mutual organizations, particularly, in the matter of expenses of members during life, and the security afforded their families after death, considerable pecuniary advantage is found to develop in favor of the latter. This will not appear strange, and in fact could hardly have been otherwise, when the high salaries paid the officers and managers at the home establishments; the expensive buildings, erected or purchased for their occupancy and use; advertising; large fees to general and local agents, etc., etc., are compared with the *inexpensiveness* of the latter organizations. In both instances, the money is derived, of course, from the assured primarily, and the premiums subsequently paid by them constitute the main source of revenue for building up a reserve fund for the final liquidation of the policies or certificates of members. The expenses connected with many of the old companies were enormous, almost fabulous, in some cases, and yet they managed to take up their policies as they became due on the death of a member, or otherwise, as long as they continued operations. In such organizations as the Independent Order of Heptasophs, Royal Arcanum, Knights of Honor, etc., the cost of the same amount of insurance, as in the older companies, is almost *nominal* in amount, when contrasted with the large premiums demanded by the latter, to perpetuate membership. The reason for this is obvious when it is recollected that the above mentioned, and others that might be named, have no salaried officials to support and make rich with high rates of premium or percentage.

In some of these latter associations there are absolutely no expenses paid to officers, excepting the small fees to medical examiners, of one, or at most two dollars, in order to ascertain whether the applicant is physically qualified for membership in the society. And as each member is the assurer of his fellow, it is to the interest of all concerned to see that only good subjects are admitted. By the laws of these associations, or societies, the greatest amount of security is afforded for the payment of the certificate, or policy, on the death of a member. Technical exceptions, and the law's delay, are practically avoided, and payment of the death claim is made without unnecessary loss of time to the party or parties for whom the benefit was intended by the member. Physicians, as a class, and the same may be said of dentists, also, rarely accumulate large estates, even from a long life of practice and toil; and their families are consequently left more or less dependent upon their own exertions after they have passed away! Would not societies for the protection of those whom they love, and desire to provide for, be attended with equally beneficial results to the families of these professional men, if arranged upon a plan similar to those thus briefly mentioned? The object is at present to direct attention to the importance of the subject, as more will be said in regard to it in future.

ADVERTISERS will find this journal peculiarly well suited for making their business known to purchasers in the medical and dental professions throughout this entire country, as its large circulation, commencing, as it does, with several thousand, will be read by professional men from Canada to Mexico, and from the Atlantic to the Pacific Ocean, and also in foreign countries. There is every reason to believe that the present large edition will be more than doubled during the first year; and that it will not only find its way into every town and hamlet of this continent, but be circulated to a considerable extent in Europe also.

COMMUNICATIONS and contributions on medical and dental science, are respectfully solicited from every quarter, as it is the wish of the editors to render it as nearly an *original* journal as possible. Please let us have contributions on all matters relating to discovery or improvements in science, as soon as convenient after they have been made. There are few professional men, indeed, who could not contribute something *new*, if they would only take the time to write it out. Remember, then, these pages are always open to all such as will favor us with their discoveries and observations.

